# The effects of individually ventilated cages on the respiratory systems of male and female Wistar rats from birth until adulthood

**DOI:** 10.6061/clinics/2017(03)07

**Published:** 2017-03

**Authors:** Guilherme D’Aprile Marchesi, Sônia de Fatima Soto, Isac de Castro, Thiago Guimarães Rodrigues, Henrique Takachi Moriya, Francine Maria de Almeida, Rogerio Pazetti, Joel Claudio Heimann, Luzia Naôko Shinohara Furukawa

**Affiliations:** I Faculdade de Medicina da Universidade de São Paulo, Laboratório de Hipertensão Experimental, Departamento de Medicina Interna, São Paulo/SP, Brazil; II Universidade de São Paulo, Escola Politécnica, Laboratório de Engenharia Biomédica, São Paulo/SP, Brazil; III Hospital das Clínicas da Faculdade de Medicina da Universidade de São Paulo, Instituto do Coração (InCor), Laboratório de Pesquisa de Cirurgia Torácica - LIM61, Departamento de Cardiopneumologia, São Paulo/SP, Brazil

**Keywords:** Individually Ventilated Cage, Sex, Rat, Development, Respiratory System

## Abstract

**OBJECTIVE:**

To evaluate the respiratory systems of male and female rats maintained in individually ventilated cages (IVCs) from birth until adulthood.

**METHODS:**

Female Wistar rats were housed in individually ventilated cages or conventional cages (CCs) and mated with male Wistar rats. After birth and weaning, the male offspring were separated from the females and kept in cages of the same type until 12 weeks of age.

**RESULTS:**

The level of food consumption was lower in male offspring (IVC=171.7±9; CC=193.1±20) than in female offspring (IVC=100.6±7; CC=123.4±0.4), whereas the water intake was higher in female offspring (IVC=149.8±11; CC=99.2±0) than in male offspring (IVC=302.5±25; CC=249.7±22) at 11 weeks of age when housed in IVCs. The cage temperature was higher in individually ventilated cages than in conventional cages for both male (IVCs=25.9±0.5; CCs=22.95±0.3) and female (IVCs=26.2±0.3; CCs=23.1±0.3) offspring. The respiratory resistance (IVC=68.8±2.8; CC=50.6±3.0) and elastance (IVC=42.0±3.9; CC=32.4±2.0) at 300 µm/kg were higher in the female offspring housed in ventilated cages. The ciliary beat values were lower in both the male (IVCs=13.4±0.2; CC=15±0.4) and female (IVC=13.5±0.4; CC=15.9±0.6) offspring housed in individually ventilated cages than in those housed in conventional cages. The total cell (IVC=117.5±9.7; CC=285.0±22.8), neutrophil (IVC=13.1±4.8; CC=75.6±4.1) and macrophage (IVC=95.2±11.8; CC=170.0±18.8) counts in the bronchoalveolar lavage fluid were lower in the female offspring housed in individually ventilated cages than in those housed in conventional cages.

**CONCLUSIONS:**

The environmental conditions that exist in individually ventilated cages should be considered when interpreting the results of studies involving laboratory animals. In this study, we observed gender dimorphism in both the water consumption and respiratory mechanics of rats kept in ventilated cages.

## INTRODUCTION

Animals used in scientific research are typically maintained in polypropylene opaque 1 or polycarbonate 2 conventional cages (CCs). Currently, these cages are being replaced with individually ventilated cages (IVCs) 2, which were designed to provide better conditions for animal welfare and decrease the workload of animal facility technicians 2. Moreover, IVCs also ensure lower pathogen transmission between animals and between animals and technicians 2. The constant air exchange inside the cages is responsible for the low levels of ammonia, CO_2_ and humidity 1-3, which can contribute to the development of respiratory diseases 4,5. The air insufflated into each IVC passes through a high-efficiency particulate arrestance (HEPA) filter installed outside of the cage, which reduces the particle concentration inside the cage. An additional benefit of IVCs is the high degree of allergen protection they afford compared with CCs 2.

The blood pressure (BP) and heart rate (HR) may be influenced by the air change rate in IVCs. Indeed, when this rate exceeds 80 air changes per hour (ACH), it is associated with increased HR and systolic BP in rats 2. In contrast, a ventilation rate of 30 ACH with weekly bedding changes provides an adequate microenvironment and avoids increases in systolic BP and HR values 2.

Laboratory animals housed in CCs are more vulnerable to inflammatory airway diseases 2. The mucociliary system plays an important role in defending against infections and inflammatory conditions by continuously removing inhaled particles, such as pollutants; pathogenic microorganisms, including bacteria and viruses; antigens; and toxins, from the external environment, which become arrested in the mucus on the surface of the tracheobronchial airway 6. The mucociliary clearance involves two principal elements that interact to clean the respiratory system: mucus and cilia. Dysfunction of the mucociliary system in response to various agents, such as drugs 7-10 and air contaminants 3,11, may be an early indicator of the inflammatory process.

Most previous studies were conducted with adult animals, and no study has evaluated animal development in IVCs from birth to adulthood.

Therefore, this study was performed to determine the influence of housing conditions on body weight, food and water intake, BP, and mucociliary and respiratory system function in rats from the prenatal period until adulthood.

## MATERIALS AND METHODS

The experimental protocol was approved by the Research Ethics Committee of the Faculdade de Medicina da Universidade de São Paulo (certificate number 036/14).

### Dams and offspring

Adult female Wistar rats from the Central Animal Facility of the School of Medicine of the University of São Paulo, São Paulo, Brazil were housed in IVCs (model ALERKS-56, Alesco Indústria e Comércio Ltda, Monte Mor, SP, Brazil) or CCs with a 12-hour light-dark cycle at 20-22°C and 50-55% humidity. The air circulation in each IVC was electronically controlled, and the air change rate was set to 30 ACH. The air inlet is located in the inferior portion of the cage. Adult female rats housed in both types of cages were mated with male Wistar rats, and pregnancy was confirmed by the presence of sperm in the vaginal fluid. The rats were individually housed throughout gestation. After delivery, the offspring remained housed in the same cage as the mother until weaning at three weeks of age. Thereafter, the offspring were separated by gender and kept in the same type of cage until 12 weeks of age. Four animals were kept in each cage. The weekly body mass was determined from birth, and the food and water intake was evaluated from 4 weeks of age until adulthood. The tail-cuff BP (TcBP) and HR were measured in male and female offspring housed in both types of cage at 6 and 12 weeks of age. The evaluations of the cage temperature, ciliary beat frequency, respiratory mechanics, and bronchoalveolar lavage (BAL) fluid were performed at 12 weeks of age.

### Husbandry conditions

The following husbandry conditions were the same for both IVCs and CCs. The bedding consisted of sterilized softwood shavings with a depth of 2 cm and was changed three times a week. The drinking water was filtered, changed three times a week, and available *ad libitum*. The feed was pelleted diet CR-1 manufactured by QUIMTIA (Colombo, PR, Brazil) and was available *ad libitum.* The dimensions of each IVC cage were 31x43x16 cm (floor space: 1333 cm^2^), and the dimensions of each CC cage were 32x39x16 cm (floor space: 1248 cm^2^).

### Evaluation of the body mass and food and water intake

The body mass was measured using an electronic scale designed for animals (Marte Balanças, model AS5500C, Sao Paulo – SP, Brazil). The intakes of food and water were determined by subtracting the amount of food or water remaining on the seventh day from that provided on the first day and dividing the result by the number of animals. The food and water consumption values are presented in units of g/week/rat and ml/week/rat, respectively.

### Cage temperature

The cage temperature was measured with a mercury thermometer when the rats were 12 weeks of age each day for one week. The thermometer was placed inside the cage, and the temperature was read after 5 minutes. We took care to prevent the animals from licking the thermometer. The measurements were collected during the morning of each day for seven days, and the values were averaged for each type of cage.

### TcBP and HR

TcBP and HR measurements were performed in conscious animals using a Kent RTBP2000 system and a Kent RTB001-R data acquisition system (Kent Scientific Corporation, Torrington, CT, USA). Briefly, each animal was warmed 10 to 15 min before being placed inside the restrainer with the tail exiting through the rear hatch. The occlusion cuff was slid to the base of the tail, and the pulse sensor was applied caudal to the occlusion cuff by wrapping the Velcro strap around the tail and the pulse sensor. When the pulse signal was at the proper amplitude, the automatic pump was inflated until the occlusion cuff pressure reached 250 mmHg. The pressure in the occlusion cuff was held at this level for five seconds and then slowly decreased using the deflate valve. The systolic BP and HR were detected by the acquisition system at the moment when the pulse signal reappeared during the deflation of the occlusion cuff. All animals were acclimated to the use of this method before the actual TcBP and HR measurements were collected. This acclimation process was performed one week before the actual measurements by placing each animal in the restraint cage and repeating the TcBP and HR measurements five times. The BP and HR values of each animal represent an average of at least five measurements.

### Ciliary beat frequency

After euthanasia via less than five minutes of an overdose of thiopental (100 mg/kg, intraperitoneal [i.p.]), the left primary bronchus was longitudinally opened and exposed in a petri dish placed on a microscope to observe the ciliated epithelium. Images of the ciliated bronchial epithelium were captured using a video camera and viewed on a monitor. A stroboscope with an optical fiber was placed in front of the ciliated epithelium to flash light emissions at a constant frequency. The flash frequency was adjusted such that ciliary movement was no longer perceived. This frequency was accepted as the ciliary beat frequency 10.

### Evaluation of respiratory mechanics

The animals were anesthetized by i.p. injection with a combination of ketamine (50 mg/kg) and xylazine (10 mg/kg) and intubated with an orotracheal polyethylene catheter. Then, the orotracheal tube was connected to a ventilator for small animals (flexiVent, SCIREQ, Montreal, Quebec, Canada), and the animals were mechanically ventilated (tidal volume of 10 ml/kg, respiratory rate of 90 breaths per minute, and positive end expiratory pressure of 3 cmH_2_O). With the animal in the supine position, the left jugular vein was cannulated for the administration of saline or methacholine solution. First, two recruitment maneuvers were performed to establish stable baseline respiratory mechanics to ensure a similar volume history before the experiments and to test for the occurrence of air leakage resulting from the intubation procedure (inflation with a linear increase in the volume of the lungs to 30 cmH_2_O and maintenance of that inflation for 3 seconds). If air leakage was detected, the intubation catheter was replaced. Subsequently, six evaluations were performed at 5-minute intervals: without administration (W/adm), saline (Sal), 1P, 2P, 3P and recovery (Rec). A saline solution (0.9% NaCl) was injected during Sal, and methacholine at doses of 3 µg/kg (1P), 30 µg/kg (2P) and 300 µg/kg (3P) were injected in 0.3-ml boluses via the jugular vein 30 seconds before the beginning of the 1P, 2P, and 3P measurements. The basal (W/adm) and recovery (Rec) measurement intervals were evaluated without injection. After starting each measurement interval, normal ventilation was interrupted every 30 seconds for 3 seconds to perturb the volume and collect pressure and volume data. Four perturbations (I, II, III, and IV) of the volume were performed during each measurement interval to evaluate the respiratory mechanics. The uni-compartmental model, which is divided into the respiratory resistance system (Rrs) and the elastance respiratory system (Ers), was used to analyze the data. The area under the curve (AUC) and the percent peak area (peak area/total area) were determined to evaluate the effect of the increasing doses of methacholine on resistance and elastance in both male and female animals. The results of this analysis are presented as a column graph in Figure 3.

### BAL fluid

Soon after the last measurement of the respiratory resistance, BAL fluid was collected by injecting 5 ml of phosphate-buffered saline (PBS) solution into the orotracheal tube. The BAL fluid was centrifuged at 300 × g for 10 minutes at 5°C. After centrifugation, the pellet was resuspended in 5 ml of PBS, and the total number of cells/ml was counted in a Neubauer chamber. The differential leucocyte count (300 cells/slide) was performed using Diff-Quik staining and Cytospin slides (Model Cytospin 2, Shandon Instruments, Sewickley, PA, USA). The total cell and differential leucocyte counts are presented in units of 10^3^ cells/mL. Subsequently, the animals were euthanized by an overdose of thiopental (100 mg/kg, i.p.).

### Statistical analyses

The results are given as the mean± standard error (SE). The statistical analyses of the body weight and water and food intake were performed by two-way analysis of variance (ANOVA). An unpaired Student’s t-test was used to analyze the cage temperature, BP, HR, ciliary beat frequency, and BAL fluid cytology. Two-way ANOVA was used to compare the means respiratory mechanics data of the two groups collected during each interval. *p*<0.05 was considered statistically significant. GraphPad Prism v.5.03 software (GraphPad Software Inc., San Diego, CA, USA) was used for all statistical analyses.

## RESULTS

### Body weight

**Figure 1:**
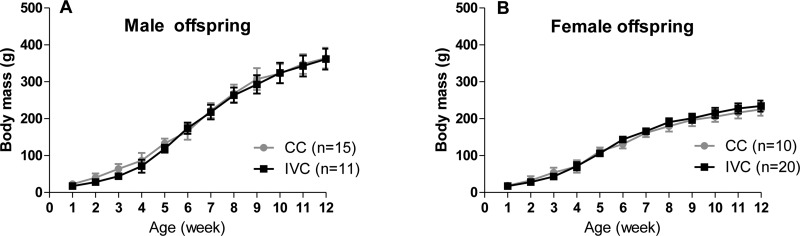
The body mass values of the male (A) and female (B) offspring housed in CCs or IVCs from 1 to 12 weeks of age. Two-way ANOVA.

The body weights of the male and female offspring did not differ between the IVC and CC groups at any point (Figure 1).

### Food and water intake

**Figure 2:**
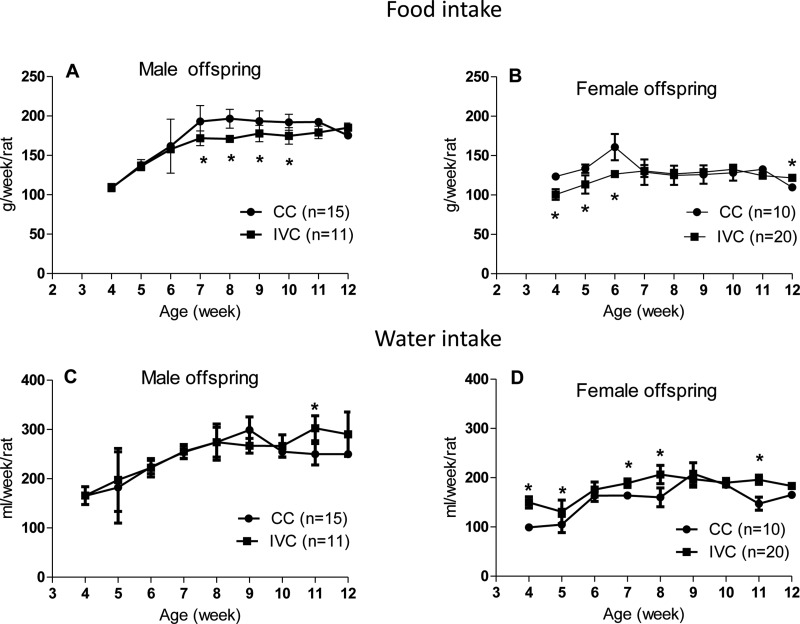
Food intake values of the male (A) and female (B) offspring and water intake values of the male (C) and female (D) offspring housed in CCs or IVCs from 4 to 12 weeks of age. *p<0.05 *vs* CC-male and *p<0.05 *vs* CC-female. Two-way ANOVA.

The food intake of the male offspring housed in IVCs was lower than that of those housed in CCs at 7, 8, 9 and 10 weeks of age. The food intake was also lower for the female offspring housed in IVCs than for those housed in CCs at 4, 5 and 6 weeks of age; however, it was higher for the female offspring in the IVCs at 12 weeks of age (Figures 2A and 2B).

The water intake was higher only in 11-week-old male offspring in IVCs relative to those housed in CCs. In contrast, the water intake of the female offspring in IVCs was higher than that of the female offspring housed in CCs at 4, 5, 7, 8 and 11 weeks of age (Figures 2C and 2D).

### Cage temperature

The cage temperature was higher (*p*<0.0001) in the IVC group than in the CC group for both male (IVCs=25.94±0.52, n=5; CCs=22.95±0.27, n=5) and female (IVCs=26.22±0.29, n=5; CCs=23.08±0.29, n=5) offspring.

### BP and HR

The TcBP and HR at 6 and 12 weeks of age did not differ between the experimental groups of both genders (Table 1).

### Respiratory system resistance and elastance

**Figure 3:**
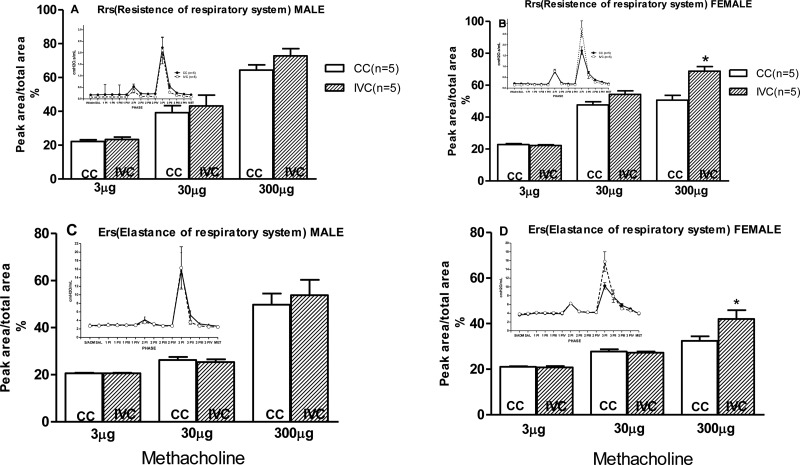
The respiratory system resistance values of the male (A) and female (B) offspring and the respiratory system elastance values in response to increasing doses of methacholine (Met) of the male (C) and female (D) offspring housed in CCs compared with those housed in IVCs evaluated by methacholine dose-response curves. W/adm - without the use of any drug or solution, Sal - saline (0.9% NaCl, 1 ml/kg), 1P - the first dose of methacholine (3 µg/kg), 2P - the second dose of methacholine (30 µg/kg), and 3P – the third dose of methacholine (300 µg/kg). Each dose was associated with 4 perturbations: PI, PII, PIII and PIV. Met measurement were performed after waiting 5 minutes for the drug to be metabolized. *p<0.05 *vs* the CC group. Two-way ANOVA.

The respiratory resistance at 300 µg/kg was higher in the female offspring housed in IVCs than in those housed in CCs (Figure 3B). Additionally, the respiratory elastance at 300 µg/kg was higher in the female offspring housed in IVCs than in those housed in CCs in response to methacholine administration (Figure 3D). No differences were observed in either the respiratory resistance or elastance in the male offspring (Figures 3A and 3C).

### Ciliary beat frequency

The ciliary beat frequencies were lower in the male and female offspring housed in IVCs than in those housed in CCs (Table 2).

### BAL fluid

The total cell, neutrophil and macrophage counts in the BAL fluid were lower in the female offspring housed in IVCs than in those housed in CCs. No differences were observed between the experimental groups of male offspring (Table 3).

## DISCUSSION

In the present study, several interesting and relevant results were found. Gender dimorphism was found to be one of the effects of housing rats in IVCs compared to CCs. In addition, housing male and female rats in IVCs was observed to increase water intake and decrease food intake. Furthermore, the internal cage temperature of IVCs was higher than that of CCs.

The lower food intake of male and female offspring housed in IVCs and absence of differences in body weight between the experimental groups indicates lower energy expenditure in IVCs. Living organisms continuously expend energy, and the rate varies with activity, ambient temperature, and many other factors 12. The lower energy expenditure can most likely be explained by the higher temperatures observed in IVCs, leading to lower food intake among the rats housed in these cages.

Water intake did not differ between the male offspring groups, whereas for female offspring, the water intake was higher in the IVC group than in the CC group throughout the developmental period. The higher cage temperature observed in IVCs than in CCs is one possible explanation for this difference, although this phenomenon did not appear to influence the male offspring. Memarzadeh et al. reported higher water intake in ventilated cages compared with CCs in a mouse study but did not address the gender of the animals or the temperature 13. It has also been observed that the temperature inside the cages tends to be higher than that outside the cages 14. Therefore, the temperature in the IVCs may be higher than that in the CCs because the lids of IVCs are closed, unlike those of CCs. As a result, dissipating the heat generated by the animals housed in IVCs is relatively difficult.

The BP and HR values did not differ between the groups, most likely because the ACH rate was adjusted to 30 ACH in this study. Previously, Krohn et al. 15 reported that air change rates exceeding 80 ACH are associated with increased HR and systolic BP in rats housed in IVCs.

In the present study, the total cell, neutrophil and macrophage counts were lower in the female offspring housed in IVCs than in those housed in CCs, whereas no differences in these counts were observed between the experimental groups of male offspring. Teixeira et al. found similar results, reporting lower total cell and macrophage counts in female rats housed in IVCs. However, they also found lower neutrophil counts in the male rats 16. Another study concluded that this difference in cell counts between males and females could be attributed to stress affecting one gender disproportionately 17. In other words, this variability in the data could be attributed to the length of the treatment and the age of the animals. In this study, the animals were kept in the same type of cage since birth. Thus, they were maintained in the same environment throughout the study. Macrophages constitute the primary defense mechanism against irritants in the respiratory tract, and their quantification is a good method of evaluating the biological-environmental conditions 7. Therefore, the female offspring most likely benefited from the microisolation provided by the IVCs, in which the ammonia concentrations, controlled air exchange rates, noise, and moisture were lower, resulting in lower rates of inflammation or infection, as noted in other studies 2,3. Interestingly, the IVC environment did not influence these cellular counts in the male offspring.

The resistance and elastance did not differ between the IVC and CC groups in the male offspring. In contrast, these parameters were higher in the female offspring in the IVC group than those in the CC group at the highest dose of methacholine (300 µg/kg). This result indicates that IVCs could contribute to maintaining a proportional responsiveness to high-dose methacholine; this effect was not observed in female offspring housed in CCs, which exhibited values comparable to those of the male offspring. Indeed, the female offspring housed in IVCs exhibited different responses to high-dose methacholine. The different responses of males and females may indicate a gender-specific effect. A previous study observed sex-specific intraperitoneal nicotine-induced asthma in rats and revealed that the methacholine-challenge response was more pronounced in male offspring than in female offspring 18.

Both male and female offspring housed in IVCs exhibited decreased ciliary beat frequencies. Alterations in the cell profile in the airways have been shown to precede significant changes in the rheological properties of the mucus 19. In the female offspring, this phenomenon could be attributed to the lower cellularity observed. However, the cellularity did not differ between the experimental groups of male offspring. Additionally, the higher cage temperature measured in the IVCs did not appear to influence the ciliary beat frequency. Indeed, in vitro and ex vivo studies have shown that the ciliary beat frequency does not change at extreme temperatures 20. Therefore, factors other than cell counts and cage temperature likely contributed to the lower ciliary beat frequency observed in the male offspring housed in IVCs.

The present study revealed that some parameters were sex specific. However, this study has some limitations, including the small sample size, the lack of other respiratory evaluation tests, and the short study period relative to the rats’ lifespan. Therefore, additional investigations are necessary because these aspects could have important ramifications for future studies.

In conclusion, the environmental conditions within IVCs should be considered when interpreting the results of studies using laboratory animals. Based on the results of this study, we observed gender dimorphism in the water consumption and respiratory mechanics of rats kept in IVCs compared to those housed in CCs.

## AUTHOR CONTRIBUTIONS

Marchesi GD performed the animal measurements, including the body weight, food and water evaluations, cage temperature, and tail-cuff blood pressure. Soto SF conducted the ciliary beat frequency measurements. De Castro I performed the statistical analysis. Rodrigues TG, Moriya HT and Pazetti R evaluated the respiratory mechanics and analyzed the results. Almeida FM collected the bronchoalveolar lavage fluid measurements. Heimann JC contributed to drafting the manuscript. Furukawa LN conceived of the study, participated in its design and coordination, and wrote the manuscript. All authors have read and approved the final version of the manuscript.

## Figures and Tables

**Table 1:** TcBP (mmHg) and HR (bpm) measured in 6- and 12-week-old male (M) and female (F) offspring housed in CCs or IVCs.

Cage type	6 weeks old		12 weeks old	
	TcBP (mmHg)	HR (bpm)	TcBP (mmHg)	HR (bpm)
CC-Male (n=14)	124.1±2.8	424.5±12.6	133.6±3.0	399.9±13.2
IVC-Male (n=11)	122.8±2.8	430.6±10.4	134.5±5.9	405.0±11.7
CC-Female (n=10)	118.7±2.3	386.3±11.5	128.8±3.1	394.1±12.8
IVC-Female (n=20)	117.9±1.6	400.9±6.06	124.5±2.4	402.2±7.6

The results are presented as the mean±SE. Student’s t-test.

**Table 2:** Ciliary beat frequencies of the male and female offspring housed in CCs or IVCs.

Type of cage	Male offspring (Hz)	Female offspring (Hz)
CC (n=5)	14.97±0.37	15.88±0.62
IVC (n=5)	13.37±0.23*	13.46±0.40**

The results are presented as the mean±SE.

* *p*<0.05 *vs* CC- male offspring;

** *p*<0.05 *vs* CC-female offspring. Student’s t-test.

**Table 3:** Total and differential leucocyte counts from BAL fluid in male and female offspring housed in CCs or IVCs.

103 Cells/ ml	Male offspring		Female offspring	
Cage	CC (n=5)	IVC (n=5)	CC (n=5)	IVC (n=5)
Total	216.25±22.02	242.5±35,5	285.0±22.8	117.5±9.7*
Neutrophils	39.71±6.7	89.8±16.5	75.6±4.1	13.1±4.8*
Lymphocytes	6.13±2.2	8.4±2.3	3.35±1.7	2.2±0.8
Eosinophils	0.22±0.22	0.0±0	0.70±0.47	1.4±0.9
Macrophages	163.4±14.5	130.±23.9	170.0±18.8	95.2±11.8*
Epithelial	6.75±2.7	14.3±4.2	4.1±4.1	5.6±4.2

The results are presented as the mean±SE.

* *p*<0.05 *vs* CC-female offspring. Student’s t-test.
